# The Bama miniature swine is susceptible to experimental HEV infection

**DOI:** 10.1038/srep31813

**Published:** 2016-08-18

**Authors:** Zi-Min Tang, Si-Ling Wang, Dong Ying, Gui-Ping Wen, Wei Cai, Ke Zhang, Wen-Fang Ji, Ming Yang, Zi-Zheng Zheng, Ning-Shao Xia

**Affiliations:** 1 State Key Laboratory of Molecular Vaccinology and Molecular Diagnostics, National Institute of Diagnostics and Vaccine Development in Infectious Diseases, School of Public Health, Xiamen University, Xiamen, Fujian 361005, PR China; 2 State Key Laboratory of Molecular Vaccinology and Molecular Diagnostics, National Institute of Diagnostics and Vaccine Development in Infectious Diseases, School of Life Sciences, Xiamen University, Xiamen, Fujian 361005, PR China

## Abstract

The hepatitis E virus (HEV) is one of the main causes of enterically transmitted hepatitis worldwide. Although the mortality rates associated with HEV are generally low, they can be up to 28% in HEV-infected pregnant women, and the elderly are more susceptible. The reasons for this selective severity are unclear, partially because there is no suitable, easy-to-use model in which to study HEV infection. Non-human primates and standard swine have been identified as being sensitive to infection with HEV and have been used for HEV infection studies. However, studies in these animals have been limited by high housing costs and the difficulty of manipulating these animals. In the current study, we established a model of HEV infection using Bama miniature swine. The model is easy to use and is sensitive to infections with HEV genotypes 3 and 4, which are classified as zoonotic HEVs. In this model, infection of Bama miniature swine with HEV genotypes 3 and 4 caused the typical features. All Bama miniature swine that were infected with HEV genotypes 3 and 4 exhibited significant HEV viremia, shedding, anti-HEV antibody responses and partial liver inflammation. Bama miniature swine may serve as an alternative to standard swine models for the study of zoonotic HEV infection and HEV genotype specificity research.

The hepatitis E virus (HEV), which causes human hepatitis E, is an important pathogen worldwide^1^,^2^,^3^. Although the mortality rate associated with this infection is generally low, it can be up to 28% among HEV-infected pregnant women^4^,^5^. At least four genotypes of mammalian HEV have been identified. HEV genotypes 1 and 2 have been found only in human beings and are responsible for a large number of water-borne epidemics in developing countries^6^. HEV genotypes 3 and 4 can infect human beings, swine and other mammalian species and can cause sporadic cases of autochthonous hepatitis E in both developing and developed countries^7^,^8^,^9^,^10^,^11^. Therefore, HEV genotypes 3 and 4 are classified as zoonotic HEVs^12^.

Although HEV was first described more than 40 years ago, research on the infection process and its pathogenesis *in vivo* remains limited because there are no available models using small, easy-to-handle animals. Non-human primates, such as cynomolgus and rhesus monkeys, are the best-known models and are susceptible to all 4 HEV genotypes. Standard swine have been experimentally infected with HEV genotypes 3 and 4, and the HEV-infected swine shed the viruses in their feces for several weeks^13^. However, studies in non-human primates and standard swine have been limited by high housing costs and difficulties involved in manipulating these animals.

Chinese Bama miniature swine are genetically stable, highly inbred, and small^14^,^15^,^16^. These animals are easier to handle than larger domestic swine. The small size of these animals makes them an ideal infection model and an attractive alternative to larger domestic swine, especially for long-term trials. Recently, specific-pathogen-free (SPF) Bama miniature swine populations have been established in China as experimental animals for medical and veterinary applications. Compared with Bama miniature swine, the cost of non-human primates was 10–20 times higher in China, and the cost of standard swine is 3–4 times higher. Additionally, experiments with non-human primates or standard swine require more personnel than experiments with Bama miniature swine. The objective of the current study was to determine whether Bama miniature swine could be suitable for use as a zoonotic HEV animal model.

## Results

**Figure 1:**
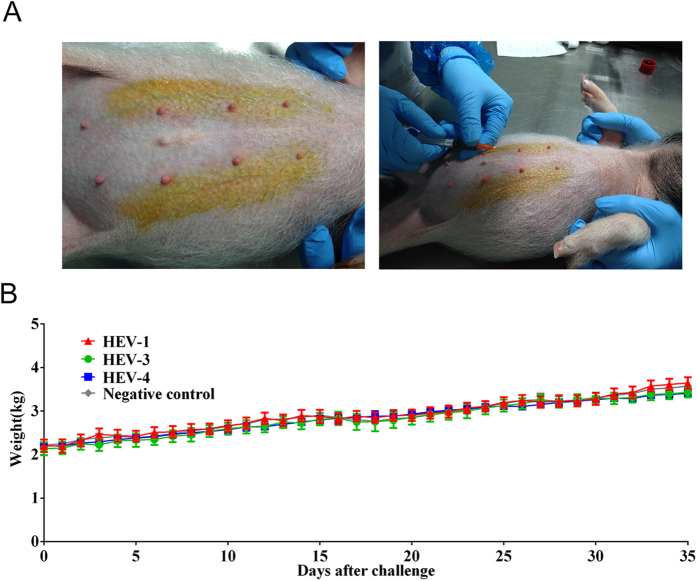
(**A**) Experimental procedure for the blood collection and virus injections via the superficial epigastric vein. (**B**) The average weights of the miniature swine after inoculation.

The miniature swine were housed separately after they were weaned. All swine were confirmed to be free of HEV through testing their feces using real-time PCR (GenMagBio, Beijing, China) and free of serum anti-HEV antibodies via testing with an HEV Ab kit (Wantai, Beijing, China) prior to inoculation. The experimental design consisted of one negative control group and three HEV-inoculated groups (see Table 1; genotypes 1, 3, and 4). Three swine were in included each HEV-inoculated group, and the swine in each of these three groups were inoculated with one of the three HEV genotypes, while two swine served as negative controls. The negative controls were housed separately and had no contact with each other. Quantification of HEV RNA in the samples was accomplished via real-time RT-PCR. The miniature swine were injected with a viral titer of 1.35 × 10^7^ genomic equivalents (GE) per mL of the viral genotype for the group to which they were assigned, and the control group swine were injected with 1 mL of phosphate-buffered saline (PBS, pH 7.4). The virus was injected into the superficial epigastric vein, administering a 1 mL volume of virus to each swine. The viral infection procedure is depicted in Fig. 1A. Each miniature swine was weighed every morning before being fed; the weights of the miniature swine after viral and control injections are presented in Fig. 1B. A linear regression analysis was performed for each group, and the weights from day 0 were normalized to 100%. The statistical analysis showed that there were no significant differences between the slopes of the curves for the different groups. The P value was 0.1591. No significant differences in weight were observed between the different groups. The average weight of the miniature swine was approximately 2 kg before inoculation. At the end of HEV shedding, the average weight of the miniature swine had increased but was still under 5 kg. This result demonstrates that HEV challenge did not negatively influence the weight of the miniature swine. After inoculation, stool samples were collected twice a week and were tested for the presence of HEV viral genomes using real-time RT-PCR and for the presence of HEV antigens using ELISA (Wantai, Beijing, China). Serum samples were collected twice per week to assess the levels of alanine aminotransferase (ALT) and total anti-HEV antibodies via ELISA (HEV-Ab kit, Wantai, Beijing, China).

**Figure 2:**
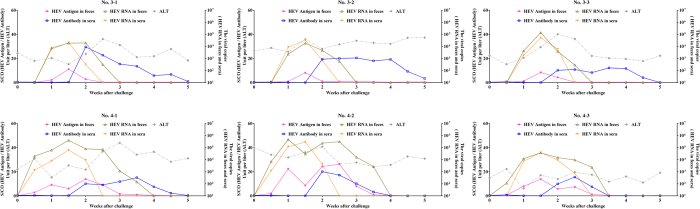
Experimental infection of Bama miniature swine with HEV genotypes 3 and 4. The variations in HEV antigens in the feces samples are shown using solid magenta squares and lines; total HEV antibodies in the sera are shown using open purple squares and lines; HEV RNA genome copies in the feces samples are indicated with open brown triangles and lines; HEV RNA genome copies in the sera are shown using solid yellow triangles and lines; ALT levels are shown with solid gray rhombuses and lines.

There were no significant elevations in ALT detected in any of the groups after inoculation. All tested parameters were negative in the genotype 1 and negative control groups, including HEV antigen, RNA in the feces and anti-HEV antibodies in the serum. These results indicate that genotype 1 of HEV failed to infect the Bama miniature swine. The data for the genotype 3 and 4 groups are shown in Fig. 2. A small elevation of ALT was observed. The pre-peak values of ALT for each swine were 1.49 (No. 3-1), 1.36 (No. 3-2), 1.80 (No. 3-3), 2.00 (No. 4-1), 1.02 (No. 4-2), and 1.48 (No. 4-3). In the genotype 3 group, tests for HEV antigen and RNA were positive 1 week after challenge and became negative 3 weeks after challenge, with viremia appearing 1 to 2 weeks after challenge. Anti-HEV antibodies were detectable in the serum samples for 2 to 5 weeks. In the genotype 4 group, HEV antigen and RNA were detected 0.5 weeks after challenge and became negative after 3 or 3.5 weeks, with viremia appearing 0.5 to 2.5 weeks after challenge. Anti-HEV antibody seroconversion was detected within 2 to 4.5 weeks after inoculation. These results clearly demonstrate that HEV genotypes 3 and 4 successfully infected the Bama miniature swine. However, no clinical symptoms were observed in the HEV-infected miniature swine. The weights of the swine that were successfully infected with HEV genotypes 3 and 4 were normal and did not differ significantly from either the negative control group or the genotype 1 group.

An additional two groups of Bama miniature swine (two 2-month-old swine per group) were challenged with 1.35 × 10^7^ copies of HEV, which was injected as previously described. Fourteen or 21 days after infection, the swine were euthanized using chloral hydrate, and obtained tissues were subjected to histopathological and immunohistochemical (IHC) examinations.

**Figure 3:**
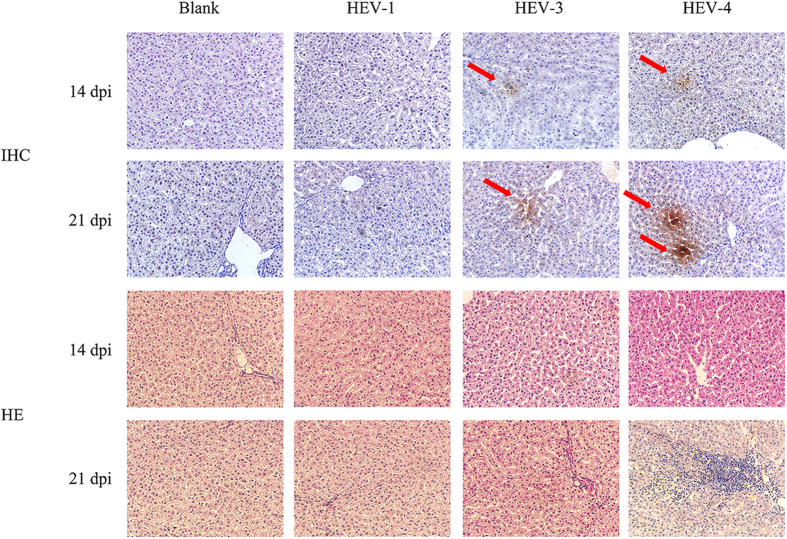
HEV antigens in the liver cells of infected swine were detected through IHC analysis and based on pathological signs of HEV infection in HE-stained liver sections. The arrows show the HEV antigens.

Through IHC staining, HEV antigens were detected in the livers of two swine that were inoculated with genotype 3 or 4 at 14 or 21 dpi, respectively. The cytoplasm of the hepatocytes from these animals stained brown, indicating that they contained HEV capsid protein (Fig. 3). No specific brown staining was observed in the livers of swine inoculated with HEV genotype 1. Genotype 4 HEV-infected swine had aggregates of lymphocytes in the hepatic lobules and exhibited mild portal inflammation. Genotype 3 HEV-infected swine displayed similar but less obvious lesions (Fig. 3). This mild hepatitis was consistent with liver enzyme levels measured in the serum. The 2 swine injected with PBS and the genotype 1 HEV-infected swine had essentially normal livers.

## Discussion

In this study, we established a Bama miniature swine HEV model. The average weight of the miniature swine was approximately 2 kg to 5 kg during infection, which made them easy to handle. Thus, Bama miniature swine could serve as an easy-to-manipulate animal for use at a model that is sensitive to zoonotic HEV (genotypes 3 and 4) infection. All of the Bama miniature swine that were inoculated with HEV genotypes 3 and 4 exhibited significant HEV viremia, shedding and anti-HEV antibody responses. The infections in this novel model were effective, and each swine showed similar results after inoculation, which may be related to the small size of Bama miniature swine, requiring lower virus titers to establish detectable infections. However, there was no prominent ALT elevation detected, as indicated by the absence of peak ALT levels above two-fold the corresponding base-line level. IHC revealed partial inflammation in the livers of the swine. IHC analysis also showed HEV antigens in the livers. These results strongly suggest that this model simulates the HEV infection process and anti-HEV antibody responses.

The reported animal models for studying HEV include various species of nonhuman primates and a number of animal models in which HEV infection is a natural occurrence, such as swine, mice, rats, chickens and rabbits. Among these animal models, the nonhuman primate model has been adapted for different HEV genotypes, but this model is expensive to maintain. Chickens and rabbits are sensitive only to avian and rabbit HEV strains, respectively. Rabbits can also be inefficiently infected by some special HEV genotype 4 strains. Mice and rats have been reported to be used as HEV animal models, but the results are inconsistent across different laboratories. Swine are the most common host for HEV other than primates, but swine can be infected only with HEV genotypes 3 and 4. Therefore, swine are widely used for research involving these genotypes. Swine models that have previously been reported have employed standard swine. Compared with standard swine, the Bama miniature swine used in the current study are more convenient to handle and exhibited more stable infections because of their smaller size and lower weight.

HEV infection exhibits distinct tropisms and different clinical characteristics in different populations. In particular, the elderly (>60 years old) are more susceptible to HEV^17^,^18^. Furthermore, the symptoms of HEV infection are more severe in pregnant women, in whom the mortality rate can be up to 28%, which is significantly higher than the average mortality rate in the rest of the population^19^,^20^,^21^. The causes underlying these phenomena are not entirely clear at present. The Bama miniature swine model used in this study provides a simple and effective research tool for further study of the HEV infection process *in vivo* as well as the pathogenic mechanisms in different HEV-infected populations.

## Materials and Methods

### Virus

The source of the viruses was stool samples from rhesus monkeys infected with genotype 1 of the virus (Xinjiang strain), genotype 3 of the virus (JRC-HE3 strain), or genotype 4 of the virus (Ch-S-1 strain). The viruses from the stool samples were diluted in PBS containing 1% bovine serum albumin (BSA) to obtain 10% (wt/vol) suspensions. These suspensions were further clarified via centrifugation at 5,000 rpm at 4 °C for 20 min and then filtered through 0.22-μm filters. HEV RNA levels in the samples were quantified using real-time RT-PCR.

### Animals

Nineteen SPF Bama miniature swine (body weight, 2–5 kg) were used in the study. The experiment was designed based on the principles outlined in the “Guide for the Care and Use of Laboratory Animals” by the National Research Council of the National Academies and the “Guide for Experimental Animal Welfare and Ethical Treatment” of the Ministry of Science and Technology of the People’s Republic of China. The experimental procedures and the animal use and care protocols were approved by the Committee on the Ethical Use of Animals of Xiamen University. The experiments were conducted in accordance with the Xiamen University Laboratory Animal Center guidelines for the use of laboratory animals. All efforts were made to minimize the numbers of animals employed and to ensure that the animals’ suffering was minimized.

### Detection of HEV RNA via quantitative reverse transcription PCR

Stool and serum samples were collected from all swine subjects twice a week, and HEV RNA was purified from 50 μl of each sample. The HEV RNA copy number was determined using quantitative real-time RT-PCR assays, as previously reported (13). A CFX96TM Real-Time System and a C1000TM thermal cycler device (Bio-Rad Inc., Hercules, CA) were used for all real-time RT-PCR assays. For the generation of standard quantification curves, (Ct) values were plotted as a function of the input HEV viral copy numbers. The copy numbers were determined by calibrating the concentration of the plasmid standard.

### Detection of HEV antigens, anti-HEV antibodies and ALT levels

After inoculation, stool and serum samples were collected twice a week. HEV antigens in those samples were detected via ELISA (Wantai, Beijing, China). The levels of ALT and the total anti-HEV antibodies in the sera were detected using HEV-Ab ELISA kits (Wantai, Beijing, China).

### Histology and IHC analysis

Livers were harvested separately and fixed through immersion in 4% formalin/PBS for 72 h at room temperature. The fixed tissues were bisected, embedded in paraffin and sectioned (4-mm thick sections). IHC analyses were performed using an Ultrasensitive^TM^ S-P kit (Fuzhou Maixin Biotechnology Development Co., Ltd., Fuzhou, China) and a DAB Detection Kit (Streptavidin-Biotin; Fuzhou Maixin Biotechnology Development Co., Ltd., Fuzhou, China) according to the manufacturer’s recommendations. The primary antibodies employed (8C11 and 15B2) were mouse anti-HEV capsid protein mAbs (1 mg/ml, 1:1,000 dilution). For histopathological analysis, tissue sections were stained with hematoxylin and eosin (HE). All sections were examined using an Olympus BH-2 microscope (Olympus, Beijing, China).

## Additional Information

**How to cite this article**: Tang, Z.-M. *et al*. The Bama miniature swine is susceptible to experimental HEV infection. *Sci. Rep.* **6**, 31813; doi: 10.1038/srep31813 (2016).

## Figures and Tables

**Table 1:** Detection of HEV antigens/RNA in the feces/HEV RNA in the serum/anti-HEV antibodies in the serum.

Inoculum	swine ID	Positive (+) or negative (−) for HEV antigens/HEV RNA detection in the feces/HEV RNA detection in the serum/anti-HEV antibody detection in the serum in the indicated week post-inoculation										
		0	0.5	1	1.5	2	2.5	3	3.5	4	4.5	5
Genotype 1	1-1	−/−/−/−	−/−/−/−	−/−/−/−	−/−/−/−	−/−/−/−	−/−/−/−	−/−/−/−	−/−/−/−	−/−/−/−	−/−/−/−	−/−/−/−
	1-2	−/−/−/−	−/−/−/−	−/−/−/−	−/−/−/−	−/−/−/−	−/−/−/−	−/−/−/−	−/−/−/−	−/−/−/−	−/−/−/−	−/−/−/−
	1-3	−/−/−/−	−/−/−/−	−/−/−/−	−/−/−/−	−/−/−/−	−/−/−/−	−/−/−/−	−/−/−/−	−/−/−/−	−/−/−/−	−/−/−/−
Genotype 3	3-1	−/−/−/−	−/−/−/−	+/+/+/−	+/+/+/−	+/+/+/+	−/+/−/+	−/−/−/+	−/−/−/+	−/−/−/+	−/−/−/+	−/−/−/−
	3-2	−/−/−/−	−/−/−/−	+/+/+/−	+/+/+/−	+/+/+/+	−/+/−/+	−/−/−/+	−/−/−/+	−/−/−/+	−/−/−/+	−/−/−/+
	3-3	−/−/−/−	−/−/−/−	+/+/+/−	+/+/+/−	+/+/+/+	−/+/−/+	−/−/−/+	−/−/−/+	−/−/−/+	−/−/−/+	−/−/−/−
Genotype 4	4-1	−/−/−/−	+/+/+/−	+/+/+/−	+/+/+/−	+/+/+/+	+/+/−/+	+/+/−/+	−/+/−/+	−/−/−/+	−/−/−/+	−/−/−/−
	4-2	−/−/−/−	+/+/+/−	+/+/+/−	+/+/+/−	+/+/+/+	+/+/−/+	+/+/−/+	−/+/−/+	−/−/−/−	−/−/−/−	−/−/−/−
	4-3	−/−/−/−	−/+/−/−	+/+/+/−	+/+/+/−	+/+/+/+	+/+/+/+	+/+/−/+	−/−/−/−	−/−/−/−	−/−/−/−	−/−/−/−
Negative control	N-1	−/−/−/−	−/−/−/−	−/−/−/−	−/−/−/−	−/−/−/−	−/−/−/−	−/−/−/−	−/−/−/−	−/−/−/−	−/−/−/−	−/−/−/−
	N-2	−/−/−/−	−/−/−/−	−/−/−/−	−/−/−/−	−/−/−/−	−/−/−/−	−/−/−/−	−/−/−/−	−/−/−/−	−/−/−/−	−/−/−/−

Samples were collected twice a week from swine that were inoculated with wild-type HEV genotype 1/3/4 or the control. The viral source was stool samples from rhesus monkeys that were infected with genotype 1 of the virus (strain Xinjiang), genotype 3 of the virus (strain JRC-HE3), or genotype 4 of the virus (strain Ch-S-1). Stool samples were tested for HEV viral genomes using RT-PCR and for HEV antigens via ELISA (Wantai, Beijing, China). Serum samples were collected to assess the levels of total anti-HEV antibodies via ELISA (Wantai, Beijing, China) and the levels of HEV RNA and HEV antigens.
